# Developing an integrated curriculum for patient safety in an undergraduate nursing program: a case study

**DOI:** 10.1186/s12912-021-00694-0

**Published:** 2021-09-17

**Authors:** Yoonjung Ji, Hyeonkyeong Lee, Taewha Lee, Mona Choi, Hyejung Lee, Sanghee Kim, Hyunok Kim Do, Sunah Kim, Sang Hui Chu, Jeongok Park, Young Man Kim, Soyoon Park

**Affiliations:** 1 Mo-Im Kim Nursing Research Institute, College of Nursing, Yonsei University, Seoul, Republic of Korea; 2 Joint Commission International, Illinois, United States; 3 College of Nursing, Research Institute of Nursing Science, Jeonbuk National University, Jeonju, Republic of Korea

**Keywords:** Patient safety, Nursing education, Curriculum, Nursing student, Competency

## Abstract

**Background:**

Nursing students’ practical training should begin when students can apply core knowledge, skills, and attitudes related to patient safety. This necessitates an integrated curriculum in nursing education that links practice to the theory concerning patient safety to enhance patient safety competencies and quality in nursing care. This study aimed to develop an integrated curriculum that incorporates patient safety factors in the existing curriculum to increase patient safety competencies in nursing students.

**Method:**

A case study approach was adopted to explain the development processes of a new curriculum integrating patient safety in the existing outcome-based curriculum of a nursing college. Based on the existing outcome-based curriculum of a nursing college, a four-step process was performed to integrate patient safety component, including quality improvement, into the curriculum: 1) literature review, 2) analysis of course syllabus, 3) selection of courses related to patient safety topics, and 4) development of evaluation tool.

**Results:**

The integrated patient safety curriculum was based on six topics: patient safety principles, teamwork, communication, patient engagement, risk management and, quality improvement, and International Patient Safety Goals. Based on the characteristics of the course according to the level of students in each year, the curriculum was integrated to address patient safety topics in seven courses (four theoretical and three practical). A Patient safety Competency self-assessment checklist was developed for students to naturally acquire patient safety competencies in clinical settings.

**Conclusions:**

This study demonstrated that patient safety topics should be addressed in both theoretical and practical settings across the entire nursing curriculum per the continuity and sequence of education principles.

**Supplementary Information:**

The online version contains supplementary material available at 10.1186/s12912-021-00694-0.

## Background

Improving the quality of healthcare systems has been gaining increased attention since the 2000s, necessitating new paradigms for quality improvement [1]. A report by the Institute of Medicine revealed that, in 2000, the number of deaths resulting from medical accidents was greater than the total number of deaths from motor vehicle accidents and HIV, leading to immense health care costs [2]. Therefore, patient safety is gaining priority in health care settings to avoid unnecessary harm to patients [3]. Patient safety involves minimizing risks and reducing exposure to mistakes and near misses during delivering health care services [2]. Over the past 10 years, efforts have been made in policy, research, and service to improve patient safety and reduce medical errors [4]. In the general industry, safety management focuses on preventing institutional financial losses [5]. Contrastingly, patient safety management in health care settings entails financial problems and the negative consequences that may occur to patients [6]; errors must not further progress into harm [7]. Therefore, health care professionals play an important role in patient safety in today’s health care environments, where improvement of patient safety and quality of care is the main outcome. For example, in Korea, the Ministry of Health and Welfare in 2018 announced the improvement of patient safety education for prospective health care professionals as part of their comprehensive patient safety plan [8]. Therefore, the increasing interest in ensuring nursing students’ competency in enhancing patient safety has been reflected in the curriculum [7]

In the 2000s, nursing education has faced numerous obstacles, which delayed education on patient safety [9]. These include a lack of awareness that patient safety training may increase patient safety skills [10], lack of confidence in preparation of curriculum and teaching methods for patient safety in nursing faculty [11], and traditional health care environments that emphasize treatment rather than disease prevention. In the U.S., the Quality and Safety Education for Nurses (QSEN) project, which started in 2005, was the first to include patient safety as an essential competency in nursing education [12]. Furthermore, in 2011, the World Health Organization (WHO) presented the guidelines on patient safety curriculum for prospective health professionals to increase their patient safety competency [13]. Since then, six competencies of QSEN—patient-centered care, teamwork and collaboration, use of the evidence-based practice, quality improvement skills, integrated use of informatics, and patient safety—have been integrated into the planning and certification standards for curriculums of nursing schools. Further, QSEN has been providing resources to increase competency in nursing academia [14]. In Canada, the Canadian Association of Schools of Nursing (CASN) and the Canadian Patient Safety Institute announced the CASN National Education Framework to provide directions and principles on interprofessional patient safety competencies for nursing students [15]. However, patient safety education is not included as a recommended or essential part of the nursing curriculum in nursing schools in Korea.

As nursing students are first exposed to clinical settings in their clinical placement, a curriculum for systemic and continuous learning of knowledge, skills, and attitudes required for patient safety must be developed before and after their clinical training. Nursing students are recommended to begin their clinical training after acquiring core knowledge, skills, and attitudes related to patient safety through theoretical classes to cope with criticism on safety incidents [16]. Therefore, content and outcomes that link theory and practice are fundamental for designing curriculums on patient safety. Previous studies on topics such as integrating quality and safety education into clinical nursing practice [17] and developing patient safety courses through literature reviews have been reported [18]; however, studies on nursing curricula are rare [19]. Additionally, patient safety education is new, contains multidisciplinary topics, such as human factors, systemic thinking, effective teamwork, and error management, which were not included in the previous program, and is contextually related to the educational content of the existing curriculum. Therefore, contextualizing the principles of patient safety across the entire curriculum is important. Thus, it is recommended that effective patient safety-related educational content is integrated into the existing curriculum rather than creating a new stand-alone course on patient safety [13]. Therefore, this study was performed to develop an integrated curriculum that assimilates patient safety elements in the existing curriculum to increase patient safety competencies in nursing students.

## Methods

This is a case study explaining the development processes of a new curriculum integrating patient safety into the existing outcome-based curriculum of a nursing college. Patient safety was newly added to core competencies in the curriculum revision stage for nursing students to respond to the needs of healthcare environment change. Accordingly, an approach that can be integrated into the current curriculum was attempted to achieve patient safety learning outcomes at the time of graduation.

The curriculum is 4 years long in the nursing college, and 126 theoretical credit hours (90 required credit hours for the major program) and 1116 h of clinical practice are mandatory. The first and second year of the curriculum include basic major courses and elective courses, simulation for clinical practice, and courses on the practice of core skills. In the third and fourth year of the curriculum, major courses are offered as an integrated curriculum based on Gordon’s functional health patterns [20]. The integrated curriculum is divided into eight domains: nutrition-metabolism-elimination, activity-rest, cognition-perception, self-concept-value/belief, roles/relationships-stress/coping, and sexuality. Each domain is integrated into courses on the nursing theory I–V and integrated nursing practice I–IV.

**Fig. 1:**
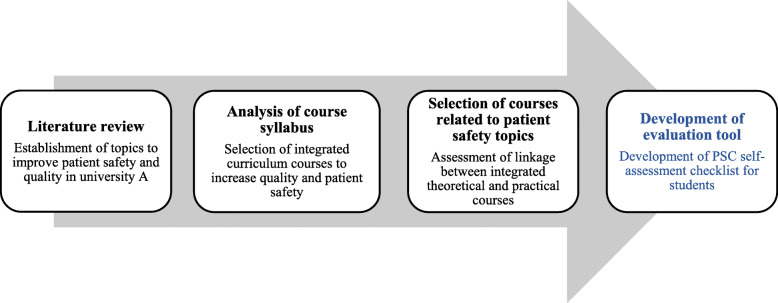
Research progress

Based on the current curriculum, a four-step process to integrate patient safety factors, as well as quality improvement, were performed in the curriculum: 1) literature review, 2) analysis of course syllabuses, 3) selection of courses related to patient safety topics, and 4) development of evaluation tool (Fig. 1).

### Literature review

The literature on patient safety competency for nursing students presented by international professional organizations was searched to select patient safety and quality improvement topics. The CASN National Education Framework [15], the WHO Patient Safety Curriculum Guide [13], and International Patient Safety Goals (IPSG) by JCI were included in the analysis [21].

### Analysis of course syllabus

The syllabus of the existing curriculum was analyzed to assess how patient safety factors were reflected in the existing curriculum and the courses in which they needed to be reflected. The learning outcomes and core topics of the syllabuses of 29 courses (24 required major courses on theory, four required major courses on practical training, and one elective major course) from all the courses offered from the first to the fourth year of the program were analyzed. First, courses related to patient safety and quality improvement were listed. Second, curriculum committee meetings were held, and experts were consulted for advice. Finally, an agreement on the curriculum improvements was reached through workshops with professors.

### Selection of courses related to patient safety topics

The courses identified by analyzing the syllabuses were classified as theoretical and practical courses, and the patient safety topics were selected, considering the students’ level each year. Experts were consulted to set the range and topics of patient safety related to the selected topics in each course. At the faculty workshops, the linkage of one of the program outcomes of the curriculum—“improvement of patient safety and quality”—and two performance criteria—“students can explain the principles of patient safety and quality improvement.” Furthermore, “students can perform nursing practice according to guidelines and procedures for improving patient safety, and quality”—with the identified theoretical and practical courses were reviewed. Further, discussions were held regarding its effectiveness in developing competency in patient safety and quality among the students.

### Development of evaluation tool

A patient safety competency checklist (PSC Checklist) was developed to enable students to enumerate the essential patient safety principles applicable in clinical practicum and self-evaluate their performance. A literature review was performed, and patient safety experts were consulted. Additionally, the PSC Checklist was reviewed by education experts, including clinical instructors and faculty members, and underwent language editing. A content validity test was also conducted by 16 teaching assistants with more than 2 years of clinical experience as a clinical instructor. Each item was assessed on a four-point Likert scale where 4 and 1 points denoted “highly valid” and “not valid at all,” respectively. If the item was rated as valid, it was classified into three types of practice level: observation, performance, and discussion. Moreover, two faculty workshops were held to prepare the final version of the checklist by reviewing the validity of the checklist items and discussing the appropriate implementation method. Lastly, the final version of the PSC Checklist was reviewed by an expert in Korean with previous experience in language editing to convey the intended meaning to the students accurately.

## Results

### Establishment of patient safety topics

**Fig. 2:**
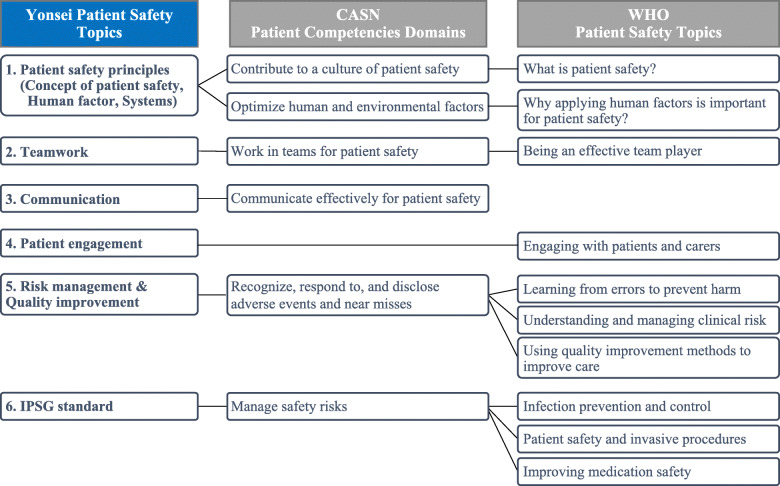
Essential patient safety topics

The analysis revealed six topics on patient safety based on the six patient safety competency domains proposed by CASN, 11 educational topics suggested by the WHO, and IPSG by JCI (Fig. 2). For example, WHO’s “what is patient safety” and CASN’s “contribute to a culture of patient safety” were derived as “concept of patient safety,” one of the sub-topics of patient safety principles, the patient safety topic of this university. By integrating WHO’s “Why applying human factors is important for patient safety?” and CASN’s “Optimize human and environmental factors,” the topics were derived as “Human factor” and “Systems” among the sub-topics of patient safety principles. Consequently, first, the sub-topics of patient safety principles consist of concepts, human factors, and systems. Contents on basic concepts were included in addition to human factors and systems that affect patient safety to improve the understanding of students who are new to patient safety. Second, information on organizing and working as a team for patient safety through teamwork and how to form multi-disciplinary cooperation works were included. Third, information on the definition of communication for patient safety and ways to communicate were included. Fourth, engaging patients and families in the treatment process were presented as a strategy to prevent accidents. Fifth, the overall process of identifying factors that harm patient safety and strategies to improve and resolve it were included for risk management and quality improvement. Lastly, the IPSG from JCI was also included. In the case of this university, a clinical practice course is provided from the third year (for 1 year) at a JCI-certified medical institution. Thus, IPSG, which can be repeatedly learned in various settings according to the curriculum’s principle of continuity and integration, has been added as the educational content. Safety guidelines related to infection, invasive behavior, and drugs, which can directly affect patient safety, were included to develop coping skills for handling various situations that may occur in clinical practice. Through the above process, patient safety topics presented by credible organizations such as WHO, CASN, and JCI were integrated into the university’s patient safety topics.

### Selection of integrated curriculum courses for patient safety competency

Based on the course characteristics according to the students’ level each year, which were obtained by analyzing existing syllabuses, the curriculum was revised to address patient safety topics in a total of seven courses. This included four theoretical (one for each grade) and three practical (one for the third year and two for the fourth year). In theoretical courses, the basic concepts of patient safety and patient engagement were introduced for approximately 3 hours in Understanding of Nursing, the first major course in the program’s first year. Understanding that patient safety is the most basic nursing concept positively affects students’ performance concerning patient safety. Moreover, the most important aspect of patient safety is patient engagement must be acknowledged from the start of the curriculum. The assignment of patient safety topics within these courses was considered regarding the level of students with a relatively low understanding of the major. Wherein allowing them to naturally acquire knowledge of the course by encountering the most basic patient safety subject from the moment they entered the nursing college.

Of the second-year courses, there was Introduction to Clinical Nursing Practice fosters knowledge and skills in basic nursing techniques before clinical training. It was modified to offer a one-hour training on the rationale and methods for considering human factors in patient safety accidents and another one-hour training on IPSG, which together form the core of basic nursing. Among the third-year courses, Communication II, an intensive communication course, was revised to offer a two-hour lecture module on communication in a team to ensure patient safety. The consensus among nursing faculty was carried out through several faculty meetings to establish a linkage between patient safety topics and courses. For example, teamwork, mainly taught in the Nursing Management course, was added to the Communication II course because communication is the most important capability in teamwork for patient safety. Moreover, the Nursing Management course in the fourth year was revised to include a six-hour module on risk management and quality improvement. Thus, the curriculum was re-organized to include elements of patient safety and the overall contents of the existing courses.

Among the practical courses, Integrated Nursing Practice I and II, which are third-year practical courses, were modified to include a 1.5-h special lecture on patient safety principles, patient engagement, and IPSG standards before clinical training. This course was modified to help students understand why patient safety is systemically important in hospitals, why patient engagement is important for patient safety, and the basic safety rules, essential for patient safety. The special lecture is provided to third-year students who are new to clinical practicum before they experience the field to remind them about patient safety topics to enhance the integration of theory-practice and safety accident prevention and coping competency. The fourth-year students, who have adapted to the basic clinical environment through their training in the third year, are prospective nurses who have to perform patient care in teams with specialists and assistants from various hospitals. Such tasks require basic knowledge of teamwork and communication. Therefore, in Integrated Nursing Practice III and IV, which are fourth-year practical courses, teamwork and communication were reviewed through a two-hour special lecture before clinical training. Lastly, Leadership Development, a fourth-year practical course, is the capstone course of the university. Therefore, all topics on patient safety, except IPSG, were reviewed through this course to ensure that students are equipped as nurses before their clinical training (Table 1).

**Table 1:** Linkage between topics of patient safety and required major courses

Course				Learning Hours by Patient Safety Topics					
	Year	Title	Outline	A	B	C	D	E	F
Theoretical	1	Understanding of Nursing	A course offered in the first semester of the first year. It provides opportunities to learn the main concepts of nursing, history of nursing, and the range of tasks and professional values of nursing. Moreover, it helps students to prepare for their own role as professional nurses through an understanding of the nursing discipline.	2			1		
	2	Introduction to Clinical Nursing Practice I/II	This course aims to provide knowledge and skills in basic nursing techniques, including core basic nursing techniques for patients to second-year nursing students who are soon to perform their first clinical practice. Students who have completed this course can apply their knowledge and solve various health problems in patients.	1					1
	3	Communication II	This course aims to improve therapeutic communications skills with various patients in clinical settings where nurses are active and improve communication skills within groups and organizations to facilitate cooperation between professionals.		1	1*			
	4	Nursing Management	This course explains the function of planning, organization, human resource management, command, and control such that the role of a nursing manager can be efficiently performed in a diversified social and health-related environment. This course helps individuals to solve problems in nursing management, understand the characteristics of nursing, and play a professional role as a nurse for advocating patient rights.					6	
Practical	3	Integrated Nursing Practice I/II	This course allows to identify factors related to nutrition-metabolism-elimination, activity-rest, cognition-perception, self-concept-value/belief, roles/relationships-stress/coping, and sexuality-reproduction function of patients in the development cycle from birth to death in addition to health problems resulting from these factors. This course helps nursing students select the appropriate procedures to solve such health problems.	0.5*			0.5*		0.5*
	4	Integrated Nursing Practice III/IV	This course allows to identify problems that hinder the health function of individuals, families, and communities with complex and special health problems over the life cycle and solve health problems through critical thinking. This course also induces primary health management and health promotion based on the health needs of individuals, families, and local communities and foster nursing leadership in nursing students so that they can actively respond to changes with organizational management capacities.		1*	1*			
	4	Leadership Development	This study aims to prepare fourth-year students for leadership and professional roles and help them successfully transition from students to professional nurses. Moreover, this course helps students understand the scope and role of nursing in hospital settings, the leadership required according to various situations, the importance of competencies (ethical and critical decision making, co-operation with other professionals, communication, and establishment of work relations). Students also have opportunities to evaluate their leadership competencies through practical application, and the course offers desirable leadership education required to be a professional nurse.	1*	*†	*	*	1*	*

A: Patient safety principles; B: Teamwork; C: Communication; D: Patient engagement; E: Risk management & Quality improvement; F: IPSG standard

^*^ Courses where academic achievement are evaluated

† If time is not indicated, these topics were included during the practical sessions without separate lecture

### Linkage between patient safety topics and theoretical and practical courses

To assess the linkage of patient safety-related education according to the program outcomes of the curriculum, performance criteria for required and elective major courses were presented according to the performance criteria set by the program outcomes. For instance, among the two performance criteria of patient safety and quality improvement, six core courses and an elective course were linked to the performance criterion of “students can explain the principles of patient safety and quality improvement.” These are Understanding of Nursing, Introduction to Clinical Nursing Practice I, Introduction to Clinical Nursing Practice II, Communication II, Integrated Nursing Practice I/ II, Nursing Management, and Patient Safety (elective). Additionally, five core courses were linked to the performance criterion of “students can perform nursing practice according to guidelines and procedures for improving patient safety and quality:” Communication II, Integrated Nursing Practice I/ II, Integrated Nursing Practice III/IV, Leadership Development, and Clinical Reasoning. The course on Clinical Reasoning was designed to assess the achievement of all expected program outcomes at the end of the four-year nursing program; further development of courses to integrate the assessment of patient safety competence into current course outcomes is required but is not within the scope of this study.

Throughout the curriculum, patient safety and quality improvement were achieved through one course in the first year, two courses in the second year, two courses in the third year, and four courses in the fourth year. The integrated curriculum was designed to achieve the performance criteria according to the learning outcomes of patient safety and quality improvement programs through 10 courses consisting of required and elective major courses. For major courses, learning outcomes were measured through the evaluation of performance criteria.

In addition, patient safety topics suitable for the Korean health care environment were set at the level of nursing colleges to increase their utilization. The systemically linked curriculum was integrated through continuous feedback and meetings of the authors. These included a JCI consultant, who is an expert in patient safety (A), and the curriculum committee members (B, C, D, E, F, G, H, Y, and J), and an advisory committee with three experts in health care professional education. Topics on patient safety were distributed across the appropriate level each year, and these were integrated into the curriculum by linking the theoretical and practical courses.

### Development of PSC self- assessment checklist for students

A PSC checklist was developed as patient safety competencies can be integrated and experienced in clinical practice based on knowledge and attitudes acquired through the theoretical courses. The PSC checklist was designed to be used as a tool for students to acquire patient safety competencies in clinical settings naturally. The list of observation or performance items and their achievement can be the criteria for course outcome evaluation. (e.g., Students mark the PSC checklist items they observed or performed on a checklist during each practice setting and submit it at the end of the course.).

This included 47 specific patient safety items that can be applied in clinical practice, primarily derived through a literature review and consultation with patient safety experts. Content validity tests for clinical instructors on the derived items showed a scale content validity index (CVI) of .91. A total of six items, including four items with an item CVI of less than .75(a. a. write an accident/incident report, b. administer high-risk drugs according to hospital policy, c. write a report on any identified risks to patient safety, d. find out the number of falls in the ward) and two items with a corrected item to a total correlation value of less than .3 (a. accompany the newly admitted patient to understand the patient’s experience, b. fill out the checklist that the nurse uses), were excluded (In the case of items that can be replaced by role-playing, most of the respondents responded without considering the possibility of role play, so even if the score was low, it was not excluded) (see Additional file 1). Similar items were compounded of the remaining 41 items, and the authors’ consensus retained the 24 items. Each item was linked to the three performance methods (observation, conduct, and discussion) to create a checklist. Afterward, the performance methods for the 24 items of the checklist were changed to two methods—observation and conduct—in the faculty workshops.

The items went through a review process by one patient safety expert and one professor with expertise in patient safety. In this process, the “Find out how many steps are in the IV pump set up procedure” item among the 24 items was deleted to reflect the expert opinion that it is ineffective for students to learn the human factor. Therefore, the final 23 PSC checklist items were derived. Subsequently, the subject and verb of the sentences were organized consistently to help the students clearly understand the items to be observed and conducted. The contents of the PSC Checklist were reviewed and agreed upon through a curriculum workshop. Further, the final draft of the checklist was prepared through an interview with a with a PhD student in the the Korean language (Table 2). Table 3 presents the main suggestions and strategies for integrating the patient safety component into the existing curriculum.

**Table 2:** Patient safety competency checklist (PSC Checklist) for clinical practicum

Course	Learning content		Item	Performance	
				Observation	Conduct
Integrated Nursing Practice I/II	1. Patient safety principles	1) Concept of patient safety	1. The doctor must check the patient identification before treatment, and, if the doctor does not, the nurse should check the patient identification	V	
		2) System	2. Analyze the cause of errors or near miss (events that were discovered before the accident and did not harm the patient)		V
		3) Human factor	3. Check how often nurses stop and perform other tasks during the administration of medication and assess the cause		V
	4. Patient Engagement		4. Medical staffs (clinicians and nurses) actively share information with patients and guardians during rounds	V	
			5. Make eye-level contact with the patients and introduce myself to the patients before starting given tasks		V
			6. Check whether the patient participated in discharge education and understood the contents of the education		V
			7. Encourage patients to participate in education for fall prevention		V
	6. IPSG		8. Accurately identify the patient (e.g., measuring vital signs, measuring liver blood glucose, administrating medication, collecting samples, treating the patient, and providing prescribed diet)		V
			9. Make a list of high-risk drugs and other drugs that look and sound similar		V
			10. Store high-risk medications in special locations where it is specified, locked, or designated	V	
			11. Comply with cautionary measures when high-risk drugs are administered	V	
			12. When a patient arrives at the operating room, be aware of the pre-operative confirmation procedures and participate in sign-in/time-out/sign-out^a^.	V	
			13. Monitor the hand hygiene practices of the medical staff in the ward		V
			14. Fill-out fall risk assessment sheet of patients		V
Integrated Nursing Practice III/IV	2. Teamwork		15. Assess how many medical staff are involved in the care of one patient		V
			16. Participate in the medical staff rounds (doctors and nurses)	V	
	3. Communication		17. Comply with principles and procedures of oral prescription when verbal prescriptions are given over the phone	V	
			18. Review the clinical results of patients and judge if it is urgent to contact a doctor		V
			19. Report the condition of the patients to the doctors^b^		V
Leadership Development	3. Communication		20. Communicate with doctors in accordance with SBAR (situation, background, assessment, recommendation) when transferring a patient (e.g., rehabilitation, x-ray examination, transfer to another ward)	V	
	5. Risk management & Quality improvement	1) Risk management	21. Identify factors in the ward that may harm patient safety (e.g., contaminated laundry without covers in the hallway, filled syringe needles, similar-looking drugs stored close to each other in one place, slippery floors, unlabeled syringes)		V
		2) Quality improvement	22. Be aware of the activities (e.g., monitoring of events, analysis of patient complaints, and checking red signal events) and methods (e.g., clinical practice improvement, root cause analysis, failure mode and effect analysis, flow charts, cause and effect diagrams, Pareto chart) performed in the ward to improve the quality of the ward		V
			23. Plan strategies to improve the problems and check whether the improvement is based on evidence (e.g., literature, clinical guidelines, data)		V

^a^ Applicable only to students of operating room practice, not applicable to students who do not have operating room training

^b^ Items that can be replaced by role play

**Table 3:** Summarizing the main suggestions and strategies for integrating curriculum

**Steps**	**Main suggestions**	**Strategies for integrating curriculum**
1) Literature review	Confirmation of educational content consistent with the position of a credible institution (e.g., WHO, CASN and JCI)	- For example, WHO’s “what is patient safety” and CASN’s “contribute to a culture of patient safety” were derived as “concept of patient safety,” one of the sub-topics of patient safety principles, the patient safety topic of this university. By integrating WHO’s “Why applying human factors is important for patient safety?” and CASN’s “Optimize human and environmental factors,” the topics were derived as “Human factor” and “Systems” among the sub-topics of patient safety principles.
	Patient safety topics decision reflecting the characteristics of each university’s curriculum and practice environment	- In the case of this university, a clinical practice course is provided from the third year (for one year) at a JCI-certified medical institution. Thus, IPSG, which can be repeatedly learned in various settings according to the curriculum’s principle of continuity and integration, has been added as the educational content.
2) Analysis of course syllabus	Analyzing all courses offered from the first to fourth year of the program	- The syllabus of the existing curriculum needs to be analyzed to assess how patient safety factors were reflected in the existing curriculum and the courses in which they needed to be reflected.
	Selection of subjects according to students’ level of understanding and patient safety topics	- The assignment of patient safety topics within these courses was considered the level of students (e.g., The basic concepts of patient safety and patient engagement were introduced in Understanding of Nursing, which is the first major course offered in the first year of the program.)
	Establishing an organic relationship on patient safety topics by courses	- It is necessary to reach a scholarly consensus throughout the school through several faculty meetings. (e.g., In this university, teamwork was included in the Nursing Management course, but after the faculty meeting, agreed that communication the most important capability in teamwork. Therefore, it was decided to add communication content related to teamwork to the Communication theory II course)
	Providing the special lecture before starting clinical practice course	- The special lecture is provided to third-year students who are new to clinical practicum before they experience the field is to remind them about patient safety topics to enhance the integration of theory-practice and safety accident prevention and coping competency.
3) Selection of courses related to patient safety topics	The linkage between patient safety topics and theoretical and practical courses	- To assess the linkage of patient safety-related education according to the program outcomes of the curriculum, performance criteria for required and elective major courses need to be presented according to the performance criteria set by the program outcomes.
4) Development of evaluation tool	Patient safety competency evaluation through PSC checklist	- The PSC checklist is designed to be used as a tool to induce students to naturally acquire patient safety topics in the clinical environment by using them during the clinical practicum courses. Therefore, the number of performances and the achievement of the items can be the criteria for a part of the evaluation(e.g., Students mark the PSC checklist items they observed or performed on a checklist during each practice course and submit it at the end of the course.)

## Discussion

Our study identified the patient safety competency topics suitable in the Korean educational context and presented a practical case of curriculum reform incorporating these topics in the existing curriculum. The integrated patient safety curriculum consisted of six topics based on the context and environment of the curriculum that students experience for 4 years. Integrating patient safety topics into the curriculum was demonstrated, and the teaching methods within the curriculum system were suggested by presenting topics according to specific courses to the educators. Patient safety education in the undergraduate nursing curriculum was revealed to be not systematically linked within or indicated in the official curriculum [22]. To our knowledge, this is the first study to design an integrated curriculum for nursing students to achieve patient safety competencies, and the results are significant as this study provided the basis for the development of a curriculum for enhancing patient safety competencies in nursing students.

Reaching a consensus with nursing education instructors on establishing and integrating necessary patient safety topics into the curriculum is fundamental [23]. This study is significant as every professor participating in the curriculum development recognized the importance of patient safety education and integrated the topics into the existing curriculum to reach a consensus. Moreover, this study did not design a separate curriculum by limiting patient safety topics to one independent course or practical setting. Instead based on the principles of continuity and sequence of the principles of curriculum organization by Tyler [24], this study attempted to continuously provide deep and broad learning and systematically distribute patient safety topics sequentially across the curriculum. Silva et al. analyzed 13 studies through an integrative review of patient safety education methods and contents, reporting that only a few elements related to patient safety were included in the curriculums. Further, most topics were sporadically taught without linking them with the other topics [22]. There are debates about the advantages and disadvantages of a single course on patient safety competencies and integration of patient safety factors across the curriculum. However, it has been argued that every topic of patient safety needs to be dealt with in-depth throughout the curriculum for health care professionals rather than adopting a superficial approach, especially in courses that link theory and practice [25]. Therefore, instructors should first recognize and sympathize with the significance of applying patient safety competencies to the curriculum to reflect patient safety competencies in the curriculum to reform it.

Learning is mostly done in classrooms and laboratories; however, the effects of learning are seen in clinical training. Significantly, the contents and teaching methods for patient safety education vary greatly between schools [26]. Furthermore, a better understanding of patient safety and developing educational methods to facilitate this understanding must come first in undergraduate nursing education so that nursing students can perform their role as prospective nurses during clinical training [27]. Therefore, this study developed a patient safety checklist to evaluate the role of nurses in the frequently encountered patient safety situations during clinical practice. Learning patient safety and acquiring practical skills and perspectives of patient-centered treatment, starting as early as in undergraduate courses, would help nursing students adapt to clinical training.

To successfully integrate patient safety into the nursing curriculum, nursing faculty should be well-prepared and familiar with teaching modern patient safety concepts systematically. Notably, instructors who lack competence in patient safety education have been continuously criticized as hindering patient safety education [13]. The lack of patient safety competency in nursing education and among clinical professionals is reported to result from a lack of understanding on how to educate students on patient safety and incorporate patient safety principles in academic and clinical settings [28]. Although the role of nursing faculty is important for improving patient safety outcomes, previous studies have mostly focused on students [23]. Some studies have evaluated the patient safety competency of nursing faculty [29]. However, no studies have assessed the relationship with the educational outcomes of teachers through the development of educational programs to enhance patient safety competencies in nursing faculty. In particular, the Nurse Educator Core Competencies, developed by the WHO in 2016, presented “curriculum and implementation” as the second among eight core competencies of nursing educators. Thereby encouraging nursing educators to develop skills to design, implement, and monitor curriculums based on the latest educational models, principles, and appropriate evidence [30]. Thus, specific curriculums to enhance the understanding of the educational content of patient safety and appropriate teaching methods need to be developed, and continuous faculty development programs for applying such curriculums need to be provided. As the role of nursing faculty is important in improving patient safety outcomes in students, future studies that examine the influence of faculty competency in patient safety on student outcomes are recommended.

This study presented patient safety topics and methods applicable to universities by developing an integrated patient safety curriculum. However, each university has different curriculums, credit systems, and courses. Thus, revisions to the curriculum developed in this study will be necessary, and the PSC Checklist will also need to be modified to reflect differences in clinical training. Moreover, the needs of students and clinical practice education officials were not surveyed, and the opinions of the stakeholders who underwent the curriculum could not be reflected. These limitations need to be compensated for through a pilot study using the checklist. This is a methodological study that proposed a process for integrating patient safety into the existing curriculum. As the developed curriculum could not be applied to nursing students, a follow-up study to apply and evaluate the integrated patient safety curriculum is suggested.

## Conclusion

This study established patient safety topics suitable for nursing education in Korea and integrated them into the existing curriculum. The key topics include basic principles, teamwork, communication, patient engagement, risk management and quality improvement, and IPSG. These are required for patient safety in nursing students and were presented, and this study proposed developing a systematically integrated curriculum for nursing faculty. This study demonstrated that patient safety topics should be dealt with in both theoretical and practical settings across the entire nursing curriculum per the continuity and sequence of education principles. Moreover, the linkage between theory and practice was secured by creating a checklist that students can use in clinical settings. The process of reforming the curriculum has provided an opportunity to recognize the importance of including nursing faculty, students, and clinical instructors related to patient safety education in improving the quality of health care through patient safety. This study provided a foundation for integrating the curriculum of patient safety nursing education in Korea’s early stages. It is thought to help develop patient safety competency and ultimately improve patient safety performance by facilitating the clinical adaptation of new nurses.

## Supplementary Information

**Additional file 1.**

## Data Availability

All data supporting the findings of this study are available from the corresponding author on request.

## Abbreviations

CASN: Canadian Association of Schools of Nursing
CVI: Content validity index
IPSG: International Patient Safety Goals
PSC Checklist: Patient safety competency checklist
QSEN: Quality and Safety Education for Nurses
