# Supplementary material for: Developing an integrated curriculum for patient safety in an undergraduate nursing program: a case study
Source: BMC Nurs. 2021 Sep 17;20:172. doi: 10.1186/s12912-021-00694-0 (PMC8447691; doi:10.1186/s12912-021-00694-0)
Supplement: Supplementary file 1 — Additional file 1. [file 12912_2021_694_MOESM1_ESM.docx]

**Additional file 1**. PRELIMINARY VERISON of patient safety competency checklist (PSC Checklist) with CVI results

| Course | Learning content | | Item | Item  CVI | M±SD | Corrected item to total correlation | Cronbach's alpha if the item was deleted |
| --- | --- | --- | --- | --- | --- | --- | --- |
| Integrated Nursing Practice I/II | 1. Patient safety principles | 1) Concept of patient safety | 1. The doctor must check the patient identification before treatment, and, if the doctor does not, the nurse should check the patient identification | .87 | 3.50±0.73 | .32 | .93 |
|  |  |  | **2. Write an accident/incident report^ab^** | .68 | 2.88±0.71 | .07 | .93 |
|  |  | 2) System | **3. Accompany the newly admitted patient to understand the patient's experience^b^** | .81 | 3.19±0.75 | .16 | .93 |
|  |  |  | 4. Analyze the cause of errors or near miss (events that were discovered before the accident and did not harm the patient) | .87 | 3.13±0.80 | .47 | .92 |
|  |  | 3) Human factor | 5. Check how often nurses stop and perform other tasks during the administration of medication and assess the cause | 1.00 | 3.50±0.51 | .55 | .92 |
|  |  |  | 6. Find out how many steps are in the IV pump set up procedure | 1.00 | 3.63±0.50 | .48 | .92 |
|  |  |  | 7. Check if the IV pump alarm is audible and can be turned off | 1.00 | 3.69±0.47 | .53 | .92 |
|  |  |  | **8. Fill out the checklist that the nurse uses^b^** | .81 | 3.13±0.71 | .13 | .93 |
|  | 4. Patient engagement |  | 9. Medical staffs (clinicians and nurses) actively share information with patients and guardians during rounds | 1.00 | 3.81±0.40 | .69 | .92 |
|  |  |  | 10. Introduce yourself to the patient before starting nursing work | 1.00 | 3.88±0.34 | .83 | .92 |
|  |  |  | 11. Ensure that the patient understands when educating the patient | 1.00 | 3.88±0.34 | .83 | .92 |
|  |  |  | 12. Make eye-level contact with the patients when talking to the patient | 1.00 | 3.81±0.40 | .69 | .92 |
|  |  |  | 13. Participate in discharge education | 1.00 | 3.56±0.51 | .56 | .92 |
|  |  |  | 14. Ensure that the patient understands the discharge education | 1.00 | 3.69±0.47 | .40 | .92 |
|  |  |  | 15. Encourage patients to participate in education for fall prevention | .93 | 3.69±0.60 | .36 | .92 |
|  | 6. IPSG |  | 16. Accurately identify the patient (e.g., measuring vital signs, measuring liver blood glucose, administrating medication, collecting samples, treating the patient, and providing prescribed diet) | 1.00 | 3.81±0.40 | .57 | .92 |
|  |  |  | 17. Check the list of high-risk drugs | 1.00 | 3.81±0.40 | .82 | .92 |
|  |  |  | 18. Find out what test results you need to call your doctor urgently | 1.00 | 3.69±0.47 | .60 | .92 |
|  |  |  | 19. Find a list of drugs that look and sound similar | .93 | 3.75±0.57 | .85 | .92 |
|  |  |  | 20. Understand how to classify and store high-risk drugs | 1.00 | 3.75±0.44 | .62 | .92 |
|  |  |  | 21. Learn about precautions when administering high-risk drugs | 1.00 | 3.69±0.47 | .53 | .92 |
|  |  |  | **22. Administer high-risk drugs to hospitals according to policy^a^** | .68 | 3.06±1.12 | .33 | .93 |
|  |  |  | 23. Confirmation procedures are performed when the surgical patient arrives at the operating room | .93 | 3.56±0.62 | .80 | .92 |
|  |  |  | 24. Check the surgical site. | .87 | 3.56±0.72 | .64 | .92 |
|  |  |  | 25. Participate in time out | .81 | 3.25±0.77 | .65 | .92 |
|  |  |  | 26. Participate in sign out | .81 | 3.31±0.79 | .68 | .92 |
|  |  |  | 27. Fill-out fall risk assessment sheet of patients | 1.00 | 3.69±0.47 | .64 | .92 |
|  |  |  | 28. Develop a treatment plan to prevent falls | .87 | 3.56±0.72 | .60 | .92 |
|  |  |  | 29. Determine whether fall prevention interventions are evidence-based | .93 | 3.44±0.62 | .59 | .92 |
|  |  |  | 30. Do hand hygiene | 1.00 | 3.88±0.34 | .83 | .92 |
|  |  |  | 31. Monitor the hand hygiene practices of the medical staff in the ward | 1.00 | 3.88±0.34 | .83 | .92 |
| Integrated Nursing Practice III/IV | 2. Teamwork | | 32. Assess how many medical staff are involved in the care of one patient | .93 | 3.69±0.62 | .67 | .92 |
|  |  |  | 33. Check how nurses participate in rounds of medical staff (doctors and nurses). | 1.00 | 3.88±0.34 | .83 | .92 |
|  |  |  | 34. Attend patient care team meetings | .93 | 3.25±0.77 | .50 | .93 |
|  | 3. Communication | | 35. Report the condition of the patients to the doctors*^b^ | .75 | 3.06±1.18 | .20 | .93 |
|  |  |  | 36. Hand over the patient when moving*^b^  (e.g., the patient is rehabilitated, x-rayed, moved to another ward, etc.) | .75 | 3.13±1.08 | .20 | .93 |
|  |  |  | 37. Fill out the SBAR SBAR (situation, background, assessment, recommendation) when transferring a patient | .87 | 3.50±0.73 | .65 | .92 |
|  |  |  | 38. Observe the nurse taking prescriptions over the phone | 1.00 | 3.88±0.34 | .83 | .92 |
|  |  |  | 39. Do hand over*^ab^ | .68 | 3.00±0.96 | .16 | .93 |
|  |  |  | 40. Review the results of clinical tests and analyze whether the results are urgent or not | 1.00 | 3.69±0.47 | .56 | .92 |
| Leadership Development | 5. Risk management & Quality improvement | 1) Risk management | 41. Identify factors that may be a risk to patient safety in the ward | 1.00 | 3.69±0.47 | .63 | .92 |
|  |  |  | **42.** **Write a report on any identified risks to patient safety^b^** | .75 | 3.13±0.80 | .30 | .93 |
|  |  | 2) Quality improvement | **43. Find out the number of falls in the ward^ab^** | .68 | 2.88±0.88 | .26 | .93 |
|  |  |  | 44. Find out what activities are being done to improve the quality of the ward | .93 | 3.75±0.57 | .65 | .92 |
|  |  |  | 45. Find out what methods are used to improve the quality of the ward | .93 | 3.56±0.62 | .62 | .92 |
|  |  |  | 46. Evaluate whether the improvement of the problem is evidence-based | .87 | 3.19±0.83 | .40 | .92 |
|  |  |  | 47. Make a plan to improve the problem | .93 | 3.38±0.61 | .32 | .92 |

Cronbach's Alpha item total=.93; Scale content validity index=.91; M±SD of items=3.51±0.71

* Items that can be replaced by role play, a=an item CVI of less than .8, b= a corrected item to a total correlation value of less than .3, deleted items displayed in bold
